# Physicians’ Mutual Benefit Association—Second Death Benefit—Paid to Mrs. Sayres

**Published:** 1886-09

**Authors:** F. E. Daniel

**Affiliations:** Secretary and Treasurer P. M. B. A.; Office Phys. Mut. Benefit Ass’n of Texas, Austin, Texas


### PHYSICIANS' MUTUAL BENEFIT ASSOCIATION OF TEXAS—PAYMENT OF SECOND DEATH-BENEFIT.

OFFICE PHYS. MUT. BENEFIT ASS'N OF TEXAS,

AUSTIN, TEXAS, Sept. 6, 1886.

Mrs. Inda Sayres, care Dr. A. Garwood, Bastrop, Texas.

DEAR MADAM:—As Secretary of the Physicians' Mutual Benefit Association of Texas, it becomes my pleasing duty to hand you herewith, Messrs. Raymond & Co's. sight draft on New York for ONE HUNDRED AND NINE DOLLARS;—the same being the full amount paid into this office under assessment on the entire membership, for the benefit of Certificate No. 89, which was held by your late husband—our lamented brother, Dr. David Sayres.

I beg you to accept this little sum as a free will offering from our small, but devoted, brotherhood of physicians; and, at the same time, please receive our assurance of sincere sympathy in your sad bereavement, and believe me

Very truly and respectfully yours,

F. E. DANIEL, M. D.,

Secretary and Treasurer P. M. B. A.

The following is the list of those who paid the assessment for the above fund. Out of a membership of one hundred and twenty-one at the time of Dr. Sayres' death, twelve failed to respond to the call, notwithstanding they had a second notice, at the end of thirty days, and an extension of twenty days. Those twelve are suspended from the Order. We are ashamed to say that amongst them are some very well known, prominent physicians;—and we do not know how to account for it.

| | | |
|---|---|---|
| F. E. Daniel, | J. P. Coffey, | J. W. Fennell, |
| R. M. Swearingen, | W. F. Buck, | E. P. Becton, |
| J. M. Litten, | J. M. Matthews, | Q. A. Shuford, |
| W. P. Burts, | M. D. Sterrett, | J. Rowland, |
| R. H. Day, | F. T. Paine, | T. S. Burk, |
| J. H. McClintock, | M. M. Myers, | J. H. Bowers, |
| H. C. Ghent, | B. E. Hadra, | A. S. Patten, |
| J. B. Duchiene, | W. M. Powell, | A. Patten, |
| S. J. Fox, | L. H. Hardy, | Jos. Greer, |
| S. W. Shoalars, | O. C. Weller, | M. R. Denman, |
| J. W. Dupree, | J. W. Daniel, | R. Leming, |
| J. H. E. Powell, | A. D. Paulus, | R. P. Talley, |
| Geo. Cupples, | J. M. Ball, | J. A. Denson, |
| F. Herf, | W. T. Strain, | C. A. Danforth, |
| D. V. Spring, | A. M. Hill, | S. F. Styles, |
| S. F. Starley, | T. J. Bennett, | W. H. Wilkes, |
| H. W. Brown, | C. C. Francis, | A. M. Womack, |
| R. G. Williams, | J. A. Throckmorton, | W. J. Dupre, |
| C. E. R. King, | J. R. Scales, | C. Sams, |
| M. A. Taylor, | J. R. Keating, | H. M. Rather, |
| J. W. McLaughlin, | J. E. Mayfield, | J. H. T. King, |
| A. R. Kilpatrick, | T. J. Murph, | R. Westmoreland, |
| F. A. Tompkins, | A. D. Tinsley, | T. J. Watlington, |
| J. T. Webb, | C. M. Harrison, | T. G. May, |
| R. W. Reid, | W. G. Jamison, | A. Welch, |
| E. S. Tidwell, | F. R. Martin, | Jos. Getzwiller, |
| W. A. Morris, | R. S. Gregg, | S. A. Stone, |
| J. M. Ross, | J. M. Lewis, | J. T. Foster, |
| H. H. Thorp, | T. M. Wilson, | W. A. McGee, |

| | | |
|---|---|---|
| J. M. Hons, | J. H. Evans, | R. A. Taylor, |
| A. N. Perkins, | J. T. Y. Jamison, | J. B. Stinson, |
| T. C. Osborn, | J. K. Fraser, | A. W. Fly, |
| F. C. Ford, | W. T. Boyd, | J. E. Kirkley, |
| Q. C. Smith, | J. T. Field, | J. M. Frey, |
| W. H. Lancaster, | A. Wagymar, | A. S. Wolff, |
| S. A. Towsey, | W. Gluver, | M. F. Wakefield, |
| | W. L. Kirksey. | |

Total, 109.

We are pleased to report the addition of twenty-one new members since the date of Dr. Sayres' death, July 2nd—making at this date one hundred and thirty members in good standing.

